# LONELINESS, ISOLATION, AND LIVING ALONE AMONG PEOPLE WITH DEMENTIA AND THEIR CARERS: INSIGHTS FROM THE IDEAL STUDY

**DOI:** 10.1093/geroni/igz038.153

**Published:** 2019-11-08

**Authors:** Christina Victor, Elizabeth B Fauth

**Affiliations:** 1 Brunel University London, Uxbridge, Middlesex, United Kingdom; 2 Utah State University, Logan, Utah, United States

## Abstract

The IDEAL research programme is national nine-year (2014-2022) ESRC/NIHR/Alzheimer’s Society UK funded longitudinal cohort study of 1547 people with mild to moderate dementia and 1283 family members or friends who provide support and aims to identify what promotes (or inhibits) people living well with dementia and their carers and how these change longitudinally. Loneliness and/or isolation are key indicators of quality of life and living well is posited as a factor which compromises wellbeing. Loneliness was measured using both the six-item de Jong Gierveld (DJG) scale (range 0-6) and isolation by the six-item Lubben social network scale (range 0 to 30). The three presentations in this symposium use data from the baseline assessment. Clare focuses upon the 18.5% of our participants who live alone and compares them with those living with others and suggests that there are few systematic differences in terms of cognition, psychological factors and well-being between these groups. Using a score of 5+ on the DJG scale, Victor reports that for people with dementia 5 % were severely lonely, which approximates to the national norm, compared with 17% for caregivers. For social isolation people with dementia had smaller social networks (mean =15.1) and higher levels of isolation as measured by a score of 12 or less on the Lubben scale (35%) compared with caregivers (mean network size=17.1 and 18% isolated). Victor and Clare use dyad data for 1089 pairs for loneliness and 1204 for isolation demonstrating congruence of 43% for loneliness and 68% for isolation

